# Vector Abundance and Genetic Diversity of *Anopheles* Mosquitoes Collected in a Laboratory–Office Complex in Vom, Nigeria: Implications for Vector Control

**DOI:** 10.1002/puh2.70079

**Published:** 2025-07-22

**Authors:** Joshua Kamani, Sacristán Irene, Arin R. Yakubu, Falmata H. Bwala, Yaarit Nahum‐Biala, Ekene H. Nnabuife, James Budaye, Shimon Harrus, Juliane Schaer

**Affiliations:** ^1^ National Veterinary Research Institute (NVRI) Vom Plateau Nigeria; ^2^ Animal Health Research Centre National Institute for Agricultural and Food Research and Technology (INIA) Centro Superior de Investigaciones Científicas (CSIC) Valdeolmos Madrid Spain; ^3^ Koret School of Veterinary Medicine The Hebrew University of Jerusalem Rehovot Israel; ^4^ Department of Biology Humboldt University Berlin Germany

**Keywords:** *Anopheles* | *cox1* | genetic diversity | malaria | vector control | workplace

## Abstract

Malaria remains a significant threat in high‐burden high‐impact (HBHI) countries despite substantial investments in disease control. This highlights the need for more comprehensive and inclusive strategies to meet national and international targets. Although agricultural and poorly maintained environments are known for mosquito breeding, workplaces are rarely considered in conventional malaria control measures. In this pilot investigation, we assessed the presence of *Anopheles* spp. in a laboratory–office complex in Vom, Nigeria, to assess workplace malaria risk and its implications for control strategies. We conducted molecular barcoding on 74 *Anopheles* specimens targeting the mitochondrial cytochrome oxidase I gene (*cox1*). Our analyses identified *Anopheles funestus* (*n* = 29; 54.6%), *Anopheles gambiae* sensu lato (*n* = 17; 32.1%), and *Anopheles rufipes* (*n* = 6; 11.3%). Haplotype network analyses revealed 12, 8, and 6 distinct haplotypes for *A*. *funestus*, *A*. *gambiae*, and *A*. *rufipes*, respectively. Genetic divergence estimates for *cox1* sequences were ≤0.011% for *A. funestus*, ≤0.007% for *A. gambiae*, and ≤0.018% for *A. rufipes*. The detection of genetically diverse *Anopheles* vector species in an office setting underscores the potential risk of workplace malaria transmission. This pilot study provides initial evidence that workplace environments can harbor genetically diverse malaria vectors and should be considered in future surveillance and control strategies. We recommend subnational tailoring (SNT) of intervention strategies to incorporate workplace environments and public places into malaria control efforts.

## Graphical abstract

Genetically diverse *Anopheles* spp. were detected in a laboratory–office complex in Vom, Nigeria, highlighting workplace malaria risk. Molecular barcoding identified *Anopheles funestus*, *Anopheles gambiae* s.l., and *Anopheles rufipes*. These findings emphasize the need to integrate workplace environments into malaria control strategies through subnational tailoring (SNT) of interventions.

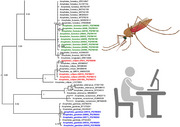

## 1 Introduction

Malaria, transmitted by various *Anopheles* mosquito species with differing bionomics, remains the most devastating human vector‐borne disease globally [1, 2, 3, 4, 5]. In malaria‐endemic regions, the disease exacerbates socioeconomic challenges, leading to poverty, malnutrition, and high mortality, particularly among children [5, 6, 7]. Consequently, malaria control and eradication efforts have received strong support from both local and international organizations [8, 9]. Although eliminating malaria would enhance resilience and foster economic growth, progress in high‐burden high‐impact (HBHI) countries, including Nigeria, remains slow, failing to meet national and international targets [5, 10].

Nigeria bears a disproportionate malaria burden, accounting for 27% of global cases and 31% of deaths [5]. Control strategies primarily focus on distribution of insecticide‐treated bed nets (ITNs), implementing indoor residual spraying (IRS), and expanding access to prompt malaria diagnosis and vaccine such as RTS, S, and R21 [5]. However, challenges such as socio‐cultural practices, logistical constrains, insecurity, and vector adaptation have hindered these efforts [11, 12]. Given malaria's complexity, tailored intervention strategies are essential. The World Health Organization (WHO) has advocated for subnational tailoring (SNT), an approach that uses local data to optimize malaria control efforts based on regional epidemiological and ecological factors [5]. Although malaria vector surveillance in Nigeria has focused primarily on agricultural and residential areas, workplace environments remain largely overlooked. Most studies document the abundance, distribution, and bionomics of dominant vectors such as *Anopheles gambiae* Giles complex and the *Anopheles funestus* Giles group in outdoor and peri‐domestic settings [2, 4]. However, malaria risk in office environments has received little attention, despite the potential for indoor mosquito exposure. Understanding the presence and genetic diversity of *Anopheles* species in workplaces is critical for effective malaria control planning.

In this pilot study, we aimed to assess the genetic diversity of *Anopheles* mosquitoes collected exclusively from a single laboratory–office complex. To achieve accurate species identification and evaluate intraspecific variation, we employed molecular barcoding using the mitochondrial cytochrome oxidase I (*cox1*) gene, a well‐established marker for mosquito taxonomy and population genetics [13, 14, 15]. Haplotype network analyses were used to assess genetic diversity within *Anopheles* species. Investigating the genetic composition of *Anopheles* populations in localized settings such as workplaces may help uncover patterns of mosquito introductions, gene flow, or population mixing that are often overlooked. Such settings could represent important, yet underappreciated, components of malaria transmission dynamics and should be considered in the context of SNT strategies aimed at optimizing vector control interventions. As a pilot study, our goal was to generate initial insights into the presence and genetic diversity of *Anopheles* mosquitoes in a workplace environment, with the aim of informing future, larger scale investigations into workplace malaria risks.

## 2 Methods

### 2.1 Study Area

The study was conducted in the Parasitology Division, National Veterinary Research Institute (NVRI) in Vom, Jos South Local Government Area (LGA), Plateau state Nigeria. The institute is located at latitude 9.733° N and longitude 8.783° E, at an altitude of 1.222 m above sea level. Vom experiences a mean annual precipitation of 1504 mm, with temperatures ranging from 13°C to 38°C. Mosquito sampling was carried out in one of two adjacent rooms within the Parasitology Division. The study room had a single door, and a large window positioned opposite the door. The window opened into a courtyard enclosed by a ∼2 m high perimeter fence. The courtyard floor consists of loose alluvial soil, which remains bare during the dry season (October–April) but is covered with grass during the rainy season (May–September). Both the door and window were fitted with fine wire mesh (<0.65 mm pore size) to restrict mosquito entry and exit. Mosquitoes observed in the passageway, on the door net, or inside the office were collected for analysis.

### 2.2 Mosquito Sampling and Identification

Mosquito sampling was conducted daily between 7:00 a.m. and 5:00 p.m. from July 2022 to December 2023. Specimens found within the office or along the corridor were captured using either an aspirator or insecticide spray. Each time a mosquito was sighted, an immediate attempt was made to capture it. Collected mosquitoes were placed in dry Petri dishes and transported to the Entomology laboratory, NVRI, Vom, for morphological identification to the genus level using taxonomic keys [16]. Each specimen was then stored individually in a coded vial containing absolute ethanol for molecular analysis. In some instances, freshly caught mosquitoes were immediately processed for DNA extraction without prior preservation.

### 2.3 DNA Extraction

Preserved mosquito specimens were individually retrieved from the ethanol and processed for DNA extraction. Each specimen was transferred to a 1.5 mL microcentrifuge tube containing sterile phosphate‐buffered saline (PBS) and washed three times by gentle manual rocking. After each wash, the PBS was removed using a sterile disposable Pasture pipette. The whole mosquito was then finely minced inside the microcentrifuge tube using the tip of a sterile 21‐gauge hypodermic needle under a dissecting microscope. DNA was extracted using the QIAamp DNA Mini Kit (QIAGEN GmbH, Hilden, Germany). Briefly, 20 µL of proteinase K and 180 µL of buffer ATL were added to each tube containing mosquito homogenate. The mixture was vortexed for 15 s and incubated at 56°C for 3 h in a heating block. Genomic DNA was then extracted according to the manufacturer's protocol. The DNA was eluted in 100 µL of elution buffer and stored at −20°C until further analysis.

### 2.4 PCR Amplification of the Cytochrome Oxidase 1 Gene (*cox1*)

Extracted DNA was used as template for conventional PCR amplification of the *cox1* gene using the standard barcoding primers LCO1490 (3′‐GGTCAACAAATCATAAAGATATTGG‐5′) and HCO2198 (3′‐AAACTTCAGGGTGACCAAAAAATCA‐5′) [17]. Each 20 µL PCR reaction contained 10 µL Phusion Flash High‐Fidelity PCR Master‐Mix (Thermo Fisher Scientific, Vilnius, Lithuania), 0.5 µL of each primer, 3 µL of template DNA, and 6 µL of nuclease‐free water (BioConcept, Allschwil, Switzerland). PCR amplification was performed on a GenAMP 7400 thermocycler (Applied Biosystems, Foster City, CA, USA). Amplicons were assessed on a 1.2% agarose gel stained with SafeView Classic (Applied Biological Materials, Richmond, BC, Canada), alongside a 100‐bp ladder (New England Biolabs, Ipswich, MA, USA), and visualized under a blue‐light transilluminator (Cleaver Scientific, UK). PCR products of the expected size (about 700 bp) were selected and sequenced at the Centre for Genomic Technologies, The Hebrew University of Jerusalem, Israel using the same primers.

### 2.5 Nucleotide Sequence Analyses

All nucleotide sequences were manually edited in the software *Geneious Prime 2022.1* (https://www.geneious.com). Ambiguous base calls were coded with the corresponding IUPAC ambiguity code, and missing data were coded as N. Sequences were aligned using the MAFFT algorithm [18, 19] implemented in *Geneious Prime*, and alignments were trimmed to obtain uniform sequence lengths of 551 bp. Sequences were compared to reference sequences on GenBank (https://www.ncbi.nlm.nih.gov/genbank/) to confirm species identity. *Anopheles* sequences of this study without ambiguous bases were used to construct haplotype networks (*n* = 51): *Anopheles rufipes*, *n* = 6; *A. gambiae*, *n* = 18; *A. funestus*, *n* = 27). Median‐joining haplotype networks for each *Anopheles* species alignment were constructed in PopART v.1.7 [20]. Analyses of evolutionary divergence estimates between individual *cox1* gene sequences within the three *Anopheles* species of the study were conducted using the Kimura 2‐parameter model [21]. The numbers of base substitutions per site between the sequences and standard error estimates were calculated (Table S1). The rate variation among sites was modeled with a gamma distribution (shape parameter = 4). Codon positions included were 1st + 2nd + 3rd. The evolutionary analyses were carried out in MEGA 7 [22]. Median‐joining haplotype networks for each *Anopheles* species with published reference sequences were carried out. Alignments were cut to shorter lengths depending on the length of the reference sequences: *A. rufipes* (*n* = 30, alignment = 431 bp), *A. gambiae* (*n* = 64, alignment = 551 bp), and *A. funestus* (*n* = 65, alignment = 448 bp). For phylogenetic analysis of the *Anopheles* species of the study, gene sequences were aligned with reference sequences (that were retrieved from NCBI GenBank) using the MAFFT algorithm [18, 19]. The alignment with 74 sequences included 20 representative *Anopheles* sequences of the study (alignment length = 606 bp). GenBank accession numbers for the reference sequences are given in the phylogenetic tree. Phylogenetic relationships were evaluated by using Bayesian inference methods. Different DNA substitution models and partition schemes were tested in PartitionFinder v.2 [23], and the best partition schemes and models were used (subsets and best model selected by BIC: Subset1 = 1–603/3 with nst = 6 = gamma; Subset2 = 2–603/3 with nst = 1 = propinv; Subset3 = 3–603/3 with nst = 6 = gamma). Bayesian inference was conducted in MrBayes 3.2.7 [24] via the CIPRES Science Gateway Web Portal V3.3 (https://doi.org/10.1109/GCE. 2010.5676129) with two runs of four chains (three heated, one cold, temperature = 0.1) each for five million generations. Bayesian support was inferred as follows: sampling every 1000 generations, convergence of runs once the value of potential scale reduction factor was between 1.00 and 1.02, and the average standard deviation of the posterior probability was <0.01. Effective sample size (ESS) was greater than 1000. The first 25% of trees were discarded as “burn‐in.” For the phylogenetic analyses, *Dixella aestivalis* was selected as the outgroup.

## 3 Results

### 3.1 Trapped Mosquito Species

A total of 313 mosquitoes were collected and morphologically identified to genus level, comprising *Aedes* spp. (*n* = 22), *Anopheles* spp. (*n* = 74), and *Culex* spp. (*n* = 217). Only *Anopheles* specimens were included in the molecular analyses. DNA extraction and *cox1* gene barcoding were performed on all 74 *Anopheles* specimens. High‐quality nucleotide sequences were obtained and subsequently analyzed for genetic diversity and phylogenetic analyses.

### 3.2 Barcoding and Haplotype Network Analyses of *Anopheles* Samples

High‐quality *cox1* sequences were obtained from 54 *Anopheles* spp. BLASTn analysis of *cox1* sequences (length 551 bp) against GenBank reference sequences identified six specimens as *A. rufipes*, 29 as *A. funestus*, and 17 as *A. gambiae* sensu lato, respectively (Table 1). One specimen (AN26) showed 100% sequence identity with *Anopheles theileri* (NCBI accession: MW603551) from Gabon (Table 1). Among the identified species, *A. funestus* was the most prevalent (54.7%), followed by *A*. *gambiae* s.l. (33.9%) and *A. rufipes* (11.3%).

**TABLE 1:** BLASTn identities of sequences of the study (551 bp).

*Anopheles* spec.	Haplotype or sample ID	BLASTn highest nucleotide identity (%)	Species according to the GenBank entry	Reference accession no.
*Anopheles funestus*	H1	99.82	*A. funestus*	MT375218
*A. funestus*	H2	99.82	*A. funestus*	OR839834
*A. funestus*	H3	99.82	*A. funestus*‐like sensu	MT917159
*A. funestus*	H4	99.82	*A. funestus*	MH299887
*A. funestus*	H5	99.82	*A. funestus*	OR839828
*A. funestus*	H6	99.82	*A. funestus*	MG742178
*A. funestus*	H7	100	*A. funestus*	OM630640
*A. funestus*	H8	100	*A. funestus*	OM630643
*A. funestus*	H9	100	*A. funestus*	MG742178
*A. funestus*	H10	100	*A. funestus*	MT375219
*A. funestus*	H11	99.46	*A. funestus*	MH299889
*A. funestus*	H12	99.82	*A. funestus*	MG742178
*A. funestus*	AN47	99.64	*A. funestus*	MH299889
*Anopheles gambiae*	H1	100	*A. gambiae*	MG930838
*A. gambiae*	H2	100	*A. gambiae*	MG753717
*A. gambiae*	H3	100	*A. gambiae*	MG753709
*A. gambiae*	H4	100	*A. gambiae*	MG930863
		100	*Anopheles coluzzii*	KR152321
		100	*Anopheles arabiensis*	MK628499
*A. gambiae*	H5	100	*A. gambiae*	MG930865
*A. gambiae*	H6	99.82	*A. gambiae*	MG753709
*A. gambiae*	H7	100	*A. gambiae*	MG753703
*A. gambiae*	H8	100	*A. coluzzii*	KR152320
*A. rufipes*	H1	99.82	*A. rufipes*	MK586051
*A. rufipes*	H2	99.27	*A. rufipes*	MK586026
*A. rufipes*	H3	100	*A. rufipes*	MK586028
*A. rufipes*	H4	99.82	*A. rufipes*	MK586028
*A. rufipes*	H5	99.82	*A. rufipes*	MK586039
*A. rufipes*	H6	100	*A. rufipes*	MK586031
*Anopheles* sp.	AN26	100	*Anopheles theileri*	MW603551

**FIGURE 1:**
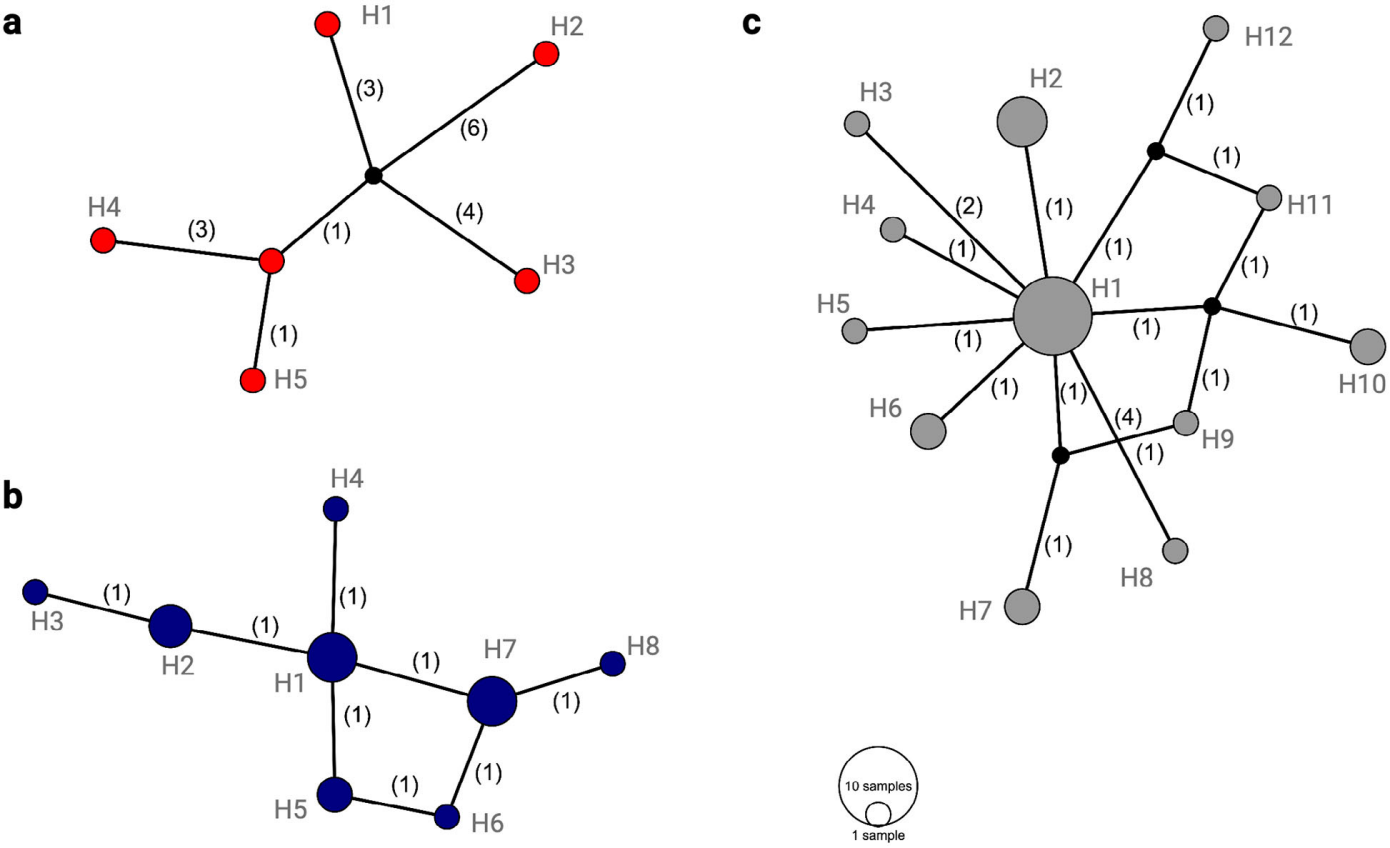
Median‐joining haplotype networks of *Anopheles* spp. based on a 551‐bp fragment of *cox1* gene for the samples sequenced in this study: (a) *Anopheles rufipes* (*n* = 6), (b) *Anopheles gambiae* (*n* = 18), and (c) *Anopheles funestus* (*n* = 27). Node size is proportional to the number of individuals assigned to each haplotype (H). A number of base changes between the haplotypes are given in parentheses. NCBI accession numbers are given in Table S2.

The haplotype network analysis for each *Anopheles* species revealed multiple distinct haplotypes among the collected specimens. Specifically, 5 *cox1* haplotypes were identified for *A. rufipes* (differing by 1–10 bp, with pairwise identity of 98.9%), 8 haplotypes for *A. gambiae* (differing by 1–6 bp, pairwise identity of 99.7%), and 12 unique haplotypes for *A. funestus* (differing by 1–6 bp, pairwise identity of 99.6%) (Figure 1a–c). Genetic divergence estimates within each species were ≤0.011% for *A. funestus*, ≤0.007% for *A. gambiae*, and ≤0.018% for *A. rufipes*. Two *Anopheles* specimens were excluded from the haplotype network and genetic distance analyses due to ambiguous base calls in their sequences. However, BLASTn analysis identified them as *A. funestus* (sample numbers: 72_AB33Q and 76_AN47). Haplotype diversity values were 0.44 for *A. funestus*, 0.47 for *A. gambiae* s.l., and 0.83 for *A. rufipes* (Table 2).

**TABLE 2:** Intraspecific comparison of *cox1* nucleotide sequences of *Anopheles* of this study.

*cox1* alignments	*A. gambiae*	*A. funestus*	*A. rufipes*
Length	551 bp	551 bp	551 bp
*n* sequences	17	27	6
Identical sites	545	535	534
Identical sites (%)	98.9	97.1	96.9
Pairwise % identity	99.7	99.6	98.9
Nucleotide diversity (π)	0.002	0.003	0.011
No. of haplotypes	8	12	5
Haplotypes diversity (Hd)	0.47	0.44	0.83

Furthermore, the intraspecific variation was assessed for the *Anopheles* species collected in Nigeria. Overall, nucleotide diversity was low across all species, with *A. rufipes* exhibiting highest diversity (*π* = 0.011), followed by *A. funestus* (*π* = 0.003). The lowest diversity was observed in *A. gambiae* s.l. (*π* = 0.002) (Table 2). Genetic divergence estimates for *cox1* sequences within each species remained low, with a maximum of 0.011% for *A. funestus*, 0.007% for *A. gambiae* s.l., and 0.018% for *A. rufipes* (Table S1).

**FIGURE 2:**
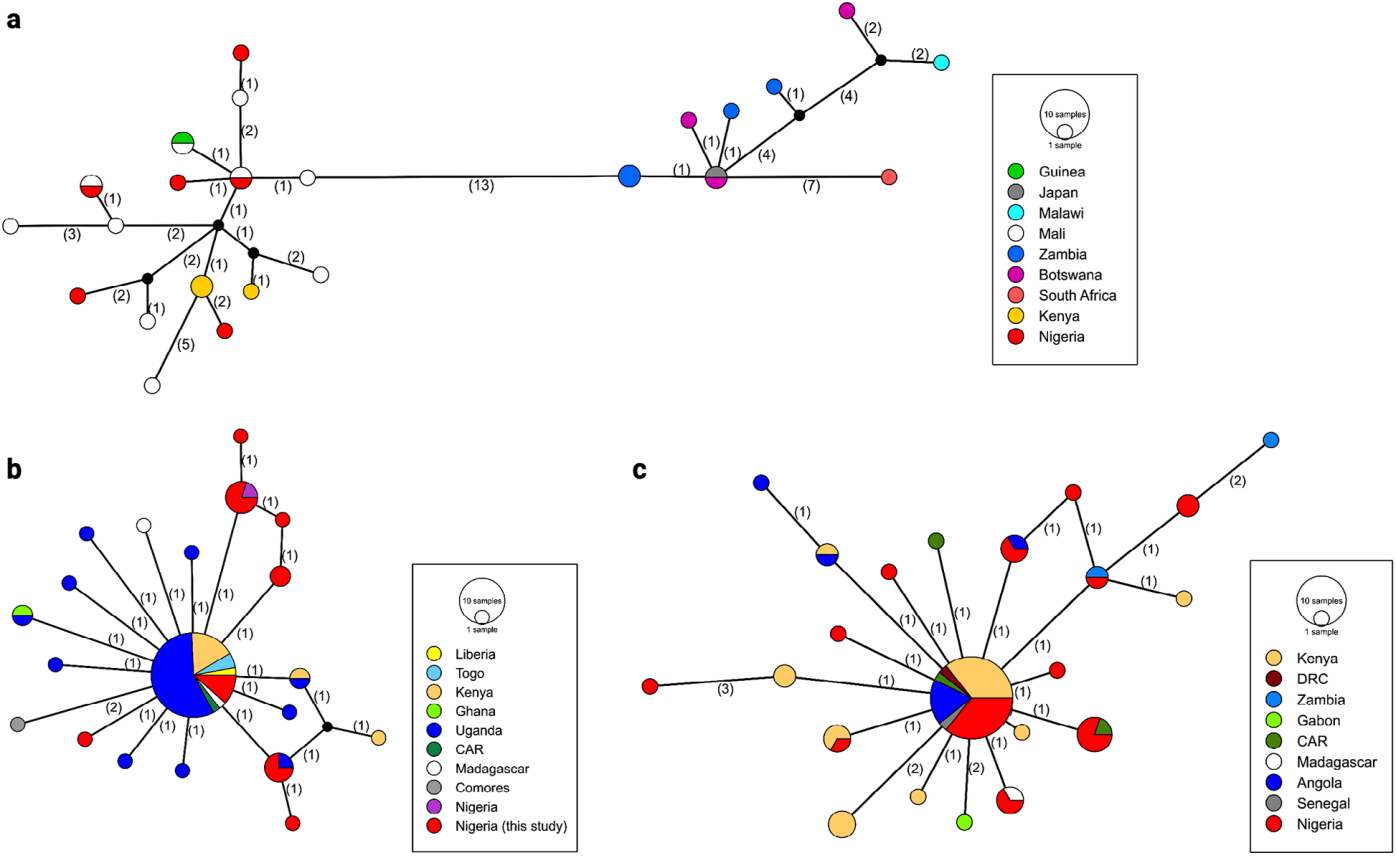
Haplotype networks of *cox1* alignments of *Anopheles* spp. of this study with reference sequences from other countries: (a) *Anopheles rufipes* (*n* = 30, alignment = 431 bp), (b) *Anopheles gambiae* (*n* = 64, alignment = 551 bp), and (c) *Anopheles funestus* (*n* = 65, alignment = 448 bp). Node size is proportional to the number of individuals assigned to each haplotype. A number of base changes between the haplotypes are given in parentheses. NCBI accession numbers of all reference sequences are given in Table S3.

Next, the nucleotide sequences obtained in this study were compared with *Anopheles cox1* sequences from other sub‐Saharan African countries to assess their genetic relatedness and potential regional patterns (Figure 2).

**FIGURE 3:**
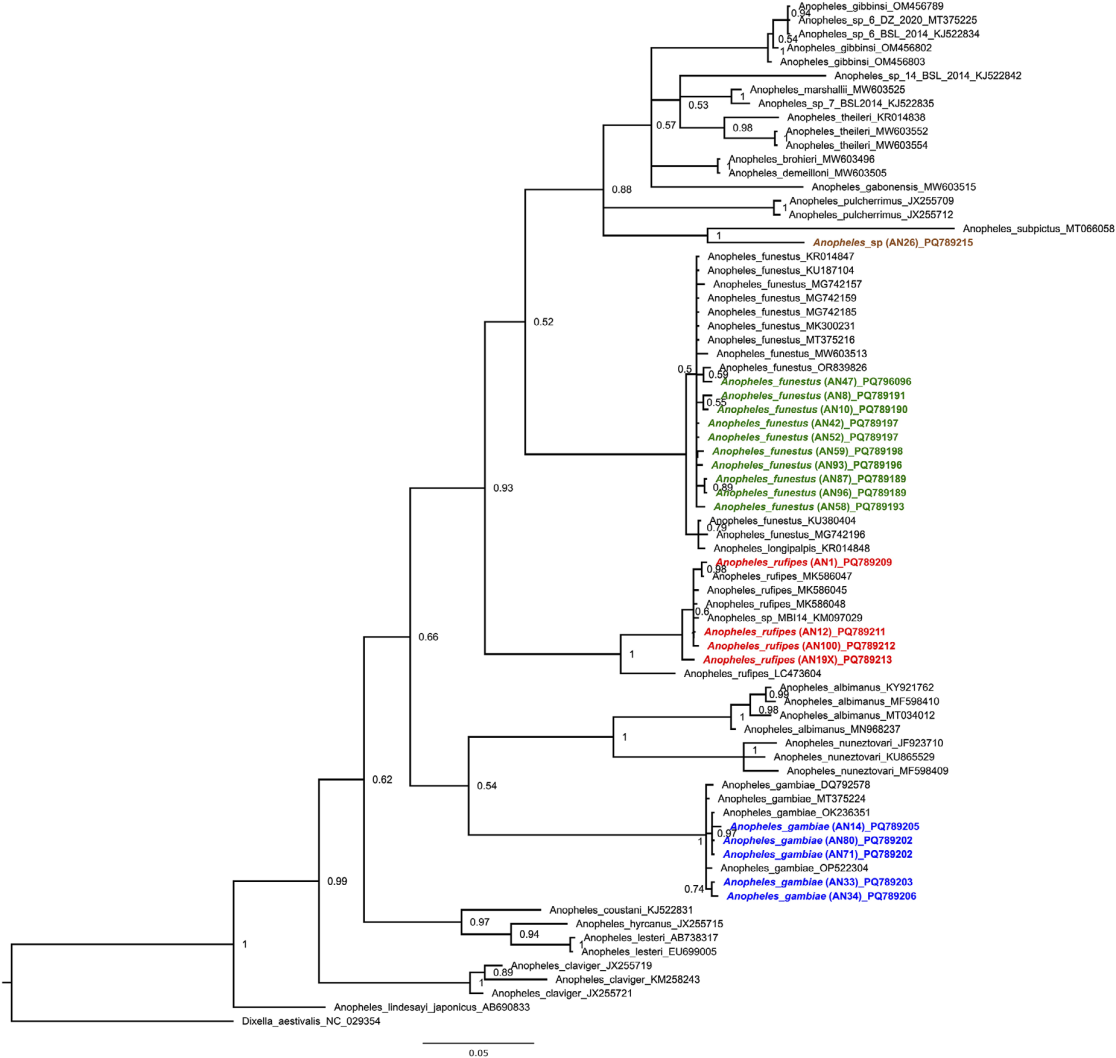
Phylogenetic relationships of *Anopheles* species. Posterior probabilities are given. The dataset included 74 *cox1* sequences and a total of 606 nucleotides (nt) without gaps. *Dixella aestivalis* was used as an outgroup. NCBI accession numbers are given in the tree. Sequences of the study are highlighted in bold and colored according to species (*Anopheles gambiae* in blue, *Anopheles rufipes* in red, and *Anopheles funestus* in grey). One sample of the study could not be assigned to a species and is therefore labeled *Anopheles* sp. (highlighted in green) and groups with sequences of *Anopheles subpictus*, *Anopheles pulcherrimus*, *and Anopheles theileri* (highest identity with NCBI accession no: MW603551, see Table 1).

The *A. rufipes* haplotypes identified in this study share two haplotypes with sequences from Mali. Additionally, the various *A. rufipes* haplotypes from this study cluster with sequences from Mali, Kenya, and Guinea, whereas a separate haplotype cluster (differing by 13 and more mutations) comprises sequences from Malawi, Zambia, South Africa, and Botswana, and a sequence entered as *A. rufipes* in GenBank from Japan (Figure 2a, Table 1). The *A. gambiae* sequences include five unique haplotypes from Nigeria, whereas two haplotypes are shared with other countries (one with Uganda and the main haplotype with multiple African countries). Notably, one haplotype is also shared with a previously published sequence from Nigeria (accession number: OK236351) (Figure 2b). Similarly, the *A. funestus* haplotype network reveals six unique haplotypes from the Nigerian samples in this study, whereas six haplotypes are shared with sequences from other African countries (Figure 2c). A Bayesian phylogenetic analysis of the partial *cox1* nucleotide sequences from this study confirmed the species identifications (Figure 3).

The *A*. *funestus* sequences formed a primary cluster with subclusters, where most sequences grouped with those from Zambia and Kenya, whereas two specimens formed a distinct subcluster with a sequence entered as *A*. *funestus* in GenBank from Portugal. A similar pattern was observed in *A. rufipes*, where sequences from this study grouped into two subclusters alongside sequences from Mali and Guinea‐Bissau. In contrast, all *A. gambiae* s.l. sequences from this study formed a single cluster with sequences from Nigeria and Kenya (Figure 3).

## 4 Discussion

Malaria remains a major public health challenge in Nigeria, despite ongoing control efforts. Since the adoption of the SNT of interventions in 2018, progress has been limited, and Nigeria continues to bear the highest malaria burden worldwide, accounting for approximately 27% of global malaria deaths and 31% of cases [5]. These statistics highlight the need for more enhanced and adaptive malaria control strategies. In addition to conventional control measures, identifying overlooked transmission risks, such as exposure in workplaces and other public settings, is essential. In this study, *Anopheles* species were opportunistically trapped in an office environment over an 18‐month period. The total of 74 specimens collected is noteworthy, particularly because mosquito abundance appeared to be correlated with human traffic in the area. *A*. *funestus* was the most frequently identified species (52.8%), followed by *A. gambiae* s.l. (33.9%) and *A. rufipes* (11.3%). The dominance of *A*. *funestus* in this indoor collection is consistent with previous studies in Nigeria, where this species has been observed resting inside human dwellings [25]. Although all specimens were collected within a single building, the presence of multiple haplotypes, particularly in *A. rufipes*, suggests cryptic population structure within species. These findings underscore the potential complexity of *Anopheles* population structure even in localized settings.

### 4.1 Genetic Diversity and Phylogenetic Relationships of the Study Species

Nucleotide sequence analyses revealed genetic diversity within each of the *Anopheles* species sampled. *A*. *funestus* exhibited multiple haplotypes with low intraspecific variation and genetic divergence, reinforcing the known species complex structure of this group [2, 26]. This aligns with previous findings that members of the *A*. *funestus* group are morphologically similar yet differ in their vector competence and ecological preferences [27]. Similarly, the *A. gambiae* s.l. specimens exhibited eight distinct haplotypes, and BLASTn searches suggested identity with *A. gambiae* s.l., *Anopheles coluzzii*, or *Anopheles arabiensis*. However, without further molecular markers (e.g., ITS2), we were unable to distinguish species within the complex. This highlights the importance of integrating multi‐marker approaches in future studies to clarify species composition, especially given the known ecological and behavioral differences among *A. gambiae* complex members.

Notably, *A. rufipes* displayed the highest haplotype diversity in this study, suggesting the presence of multiple forms or cryptic diversity, consistent with previous reports describing morphological variations in this species, including the typical form and a darker variant *A. rufipes brousseri* in Cameroon [2]. Although historically considered a secondary malaria vector due to its zoophilic tendencies, increasing evidence indicates that *A. rufipes* may contribute to malaria transmission in certain settings [28, 29, 30]. Its presence in an indoor setting further reflects its ecological adaptability. Importantly, the detection of multiple haplotypes in all three species, despite sampling from a single workplace, may indicate repeated introductions from surrounding environments or local population mixing. Similar diversity patterns have been reported in *A*. *funestus* and *A. gambiae* s.l. in other regions of Africa, reinforcing the idea that gene flow and ecological flexibility are characteristic of vector populations in Africa (e.g., [31, 32]). These findings highlight the need for fine‐scale molecular surveillance to inform targeted vector control strategies, particularly in overlooked environments such as workplaces.

### 4.2 Implications for Malaria Transmission and Control

The detection of *A*. *funestus* and *A. gambiae* s.l. in an office setting suggests that malaria vectors may be more widespread in human environments than conventionally assumed. The presence of freshly blood‐fed specimens indicates that these mosquitoes successfully obtained blood meals, raising concerns about potential transmission risks in nonresidential settings. Although this study did not assess the source of blood meals due to limited resources, previous research has demonstrated that *A*. *funestus* and *A. gambiae* are highly anthropophilic and capable of adapting their feeding and resting behaviors based on environmental conditions [4, 33].

Interestingly, the daytime collection of *A. gambiae* s.l. contradicts the widely reported preference for nocturnal feeding [34, 35]. However, this observation is consistent with prior findings that members of this species complex exhibit phenotypic plasticity, including diurnal resting behavior in indoor environments [3, 36]. Although our results suggest that *A. gambiae* s.l. may be resting indoors during the daytime, additional research is needed to determine whether this behavior is linked to recent feeding events or adaptation to environmental conditions.

The potential role of workplaces in malaria exposure deserves further investigation. Although homes remain primary sites of transmission, individuals spend considerable time in offices, schools, and other public spaces, which may serve as overlooked exposure sites. Future studies should assess mosquito abundance, biting behavior, and parasite infection rates in such settings to determine their epidemiological significance.

## 5 Conclusions

Despite numerous malaria control interventions, Nigeria continues to experience high malaria burden, highlighting the need for more targeted and adaptive strategies. This study provides evidence of malaria vectors in an office setting, reinforcing the importance of considering nontraditional exposure sites in malaria control programs. The genetic analysis confirmed the presence of *A*. *funestus, A. gambiae* s.l., and *A. rufipes*, with varying levels of haplotype diversity and genetic divergence.

## Author Contributions

**Joshua Kamani**: conceptualization, writing – original draft, funding acquisition, investigation, methodology, validation, writing – review and editing, data curation, project administration, formal analysis. **Sacristán Irene**: data curation, formal analysis, investigation, methodology, writing – review and editing. **Arin R. Yakubu**: resources, investigation, formal analysis, methodology. **Falmata H. Bwala**: investigation, resources, formal analysis, methodology. **Yaarit Nahum‐Biala**: methodology, formal analysis, investigation. **Ekene H. Nnabuife**: resources, investigation. **James Budaye**: methodology, investigation, resources. **Shimon Harrus**: writing – original draft, writing – review and editing, investigation. **Juliane Schaer**: visualization, writing – review and editing, formal analysis, validation, data curation.

## Ethics Statement

The authors have nothing to report.

## Consent

The authors have nothing to report.

## Conflicts of Interest

The authors declare no conflicts of interest.

## Supporting information



Table S1 Estimates of evolutionary divergence between sequences.
**Table S2** Haplotypes and accession numbers for sequences of the study.
**Table S3** Accession numbers and countries for reference sequences used in haplotype network analyses.

## Data Availability

Nucleotide sequence data reported in this article are available in GenBank under the accession numbers PQ789189–PQ789215, PQ796096. The datasets are partially included in this published article (and its Supporting Information). Any additional generated data and datasets used and/or analyzed during the current study are available from the corresponding authors on reasonable request.
