# Supplementary material for: Vector Abundance and Genetic Diversity of Anopheles Mosquitoes Collected in a Laboratory–Office Complex in Vom, Nigeria: Implications for Vector Control
Source: Public Health Chall. 2025 Jul 22;4(3):e70079. doi: 10.1002/puh2.70079 (PMC12282284; doi:10.1002/puh2.70079)
Supplement: Supplementary file 1 — Table S1 Estimates of evolutionary divergence between sequences. Table S2 Haplotypes and accession numbers for sequences of the study. Table S3 Accession numbers and countries for reference sequences used in haplotype network analyses. [file PUH2-4-e70079-s001.docx]

**Supplementary Information**

**Vector abundance and genetic diversity of *Anopheles* mosquitoes collected in a laboratory-office complex in Vom, Nigeria: implications for vector control**

Joshua Kamani^1*^, Sacristán Irene^2^, Arin R. Yakubu^1^, Falmata H. Bwala^1^, Yaarit Nahum-Biala^3^, Ekene H. Nnabuife^1^, James Budaye^1^, Shimon Harrus^3^, Juliane Schaer^4*^

**Supplementary Table S1. Estimates of Evolutionary Divergence between Sequences**

*Anopheles rufipes:*

| *Sample No.* | AN1 | AN32 | AN12 | AN100 | AE19X | AN80 |
| --- | --- | --- | --- | --- | --- | --- |
| AN1 |  | 0,00534 | 0,00320 | 0,00372 | 0,00537 | 0,00502 |
| AN32 | 0,01486 |  | 0,00497 | 0,00534 | 0,00602 | 0,00570 |
| AN12 | 0,00550 | 0,01296 |  | 0,00183 | 0,00416 | 0,00372 |
| AN100 | 0,00737 | 0,01486 | 0,00182 |  | 0,00459 | 0,00419 |
| AE19X | 0,01490 | 0,01867 | 0,00921 | 0,01110 |  | 0,00499 |
| AN80 | 0,01304 | 0,01678 | 0,00737 | 0,00924 | 0,01299 |  |

The number of base substitutions per site from between sequences are shown. Standard error estimate(s) are shown above the diagonal. Analyses were conducted using the Kimura 2-parameter model [1]. The rate variation among sites was modeled with a gamma di

*Anopheles gambiae:*

| *Sample No.* | 01AN9 | 03AN6 | 05AN6 | 08AN9 | 13AN33 | 15AN40 | 16AN14 | 18AN34 | 28AN53 | 35AN20 | 37AN53 | 38AN47 | 52AN139 | 59AN95 | 81AN88 | 84AN80 | 86AN71 |
| --- | --- | --- | --- | --- | --- | --- | --- | --- | --- | --- | --- | --- | --- | --- | --- | --- | --- |
| 01AN9 |  | 0,00000 | 0,00000 | 0,00000 | 0,00179 | 0,00183 | 0,00253 | 0,00260 | 0,00185 | 0,00179 | 0,00185 | 0,00189 | 0,00179 | 0,00183 | 0,00258 | 0,00183 | 0,00183 |
| 03AN6 | 0,00000 |  | 0,00000 | 0,00000 | 0,00179 | 0,00183 | 0,00253 | 0,00260 | 0,00185 | 0,00179 | 0,00185 | 0,00189 | 0,00179 | 0,00183 | 0,00258 | 0,00183 | 0,00183 |
| 05AN6I | 0,00000 | 0,00000 |  | 0,00000 | 0,00179 | 0,00183 | 0,00253 | 0,00260 | 0,00185 | 0,00179 | 0,00185 | 0,00189 | 0,00179 | 0,00183 | 0,00258 | 0,00183 | 0,00183 |
| 08AN9I | 0,00000 | 0,00000 | 0,00000 |  | 0,00179 | 0,00183 | 0,00253 | 0,00260 | 0,00185 | 0,00179 | 0,00185 | 0,00189 | 0,00179 | 0,00183 | 0,00258 | 0,00183 | 0,00183 |
| 13AN33 | 0,00182 | 0,00182 | 0,00182 | 0,00182 |  | 0,00257 | 0,00309 | 0,00185 | 0,00259 | 0,00000 | 0,00259 | 0,00258 | 0,00000 | 0,00257 | 0,00316 | 0,00257 | 0,00257 |
| 15AN40 | 0,00182 | 0,00182 | 0,00182 | 0,00182 | 0,00365 |  | 0,00171 | 0,00327 | 0,00258 | 0,00257 | 0,00258 | 0,00265 | 0,00257 | 0,00000 | 0,00185 | 0,00000 | 0,00000 |
| 16AN14 | 0,00365 | 0,00365 | 0,00365 | 0,00365 | 0,00549 | 0,00182 |  | 0,00368 | 0,00316 | 0,00309 | 0,00316 | 0,00321 | 0,00309 | 0,00171 | 0,00257 | 0,00171 | 0,00171 |
| 18AN34 | 0,00366 | 0,00366 | 0,00366 | 0,00366 | 0,00182 | 0,00549 | 0,00734 |  | 0,00308 | 0,00185 | 0,00308 | 0,00329 | 0,00185 | 0,00327 | 0,00365 | 0,00327 | 0,00327 |
| 28AN53 | 0,00182 | 0,00182 | 0,00182 | 0,00182 | 0,00366 | 0,00365 | 0,00549 | 0,00550 |  | 0,00259 | 0,00000 | 0,00264 | 0,00259 | 0,00258 | 0,00183 | 0,00258 | 0,00258 |
| 35AN20 | 0,00182 | 0,00182 | 0,00182 | 0,00182 | 0,00000 | 0,00365 | 0,00549 | 0,00182 | 0,00366 |  | 0,00259 | 0,00258 | 0,00000 | 0,00257 | 0,00316 | 0,00257 | 0,00257 |
| 37AN53 | 0,00182 | 0,00182 | 0,00182 | 0,00182 | 0,00366 | 0,00365 | 0,00549 | 0,00550 | 0,00000 | 0,00366 |  | 0,00264 | 0,00259 | 0,00258 | 0,00183 | 0,00258 | 0,00258 |
| 38AN47 | 0,00182 | 0,00182 | 0,00182 | 0,00182 | 0,00366 | 0,00365 | 0,00549 | 0,00550 | 0,00366 | 0,00366 | 0,00366 |  | 0,00258 | 0,00265 | 0,00322 | 0,00265 | 0,00265 |
| 52AN139 | 0,00182 | 0,00182 | 0,00182 | 0,00182 | 0,00000 | 0,00365 | 0,00549 | 0,00182 | 0,00366 | 0,00000 | 0,00366 | 0,00366 |  | 0,00257 | 0,00316 | 0,00257 | 0,00257 |
| 59AN95 | 0,00182 | 0,00182 | 0,00182 | 0,00182 | 0,00365 | 0,00000 | 0,00182 | 0,00549 | 0,00365 | 0,00365 | 0,00365 | 0,00365 | 0,00365 |  | 0,00185 | 0,00000 | 0,00000 |
| 81AN88 | 0,00365 | 0,00365 | 0,00365 | 0,00365 | 0,00549 | 0,00182 | 0,00366 | 0,00734 | 0,00182 | 0,00549 | 0,00182 | 0,00549 | 0,00549 | 0,00182 |  | 0,00185 | 0,00185 |
| 84AN80 | 0,00182 | 0,00182 | 0,00182 | 0,00182 | 0,00365 | 0,00000 | 0,00182 | 0,00549 | 0,00365 | 0,00365 | 0,00365 | 0,00365 | 0,00365 | 0,00000 | 0,00182 |  | 0,00000 |
| 86AN71 | 0,00182 | 0,00182 | 0,00182 | 0,00182 | 0,00365 | 0,00000 | 0,00182 | 0,00549 | 0,00365 | 0,00365 | 0,00365 | 0,00365 | 0,00365 | 0,00000 | 0,00182 | 0,00000 |  |

The number of base substitutions per site from between sequences are shown. Standard error estimate(s) are shown above the diagonal. Analyses were conducted using the Kimura 2-parameter model [1]. The rate variation among sites was modeled with a gamma di

*Anopheles funestus:*

| *Sample No.* | 07_AN8 | 09_AN10 | 14_AN42 | 19_AN37 | 20_AN38 | 21_AN41 | 30_AN52 | 31_AN60 | 32_AN59 | 44_AN50P | 46_AN111 | 47_AN133 | 48_AN129 | 49_A133X | 50_AN136 | 53_AN20 | 56_AN54 | 60_AN93 | 62_AN77 | 64_AN87 | 66_AN96 | 69_AN68 | 70_AN58 | 73_AN41Q | 74_A112R | 79_AN72 | 83_AN89 |
| --- | --- | --- | --- | --- | --- | --- | --- | --- | --- | --- | --- | --- | --- | --- | --- | --- | --- | --- | --- | --- | --- | --- | --- | --- | --- | --- | --- |
| 07_AN8 |  | 0,00264 | 0,00251 | 0,00370 | 0,00457 | 0,00320 | 0,00251 | 0,00266 | 0,00312 | 0,00251 | 0,00311 | 0,00251 | 0,00311 | 0,00251 | 0,00311 | 0,00251 | 0,00000 | 0,00320 | 0,00320 | 0,00385 | 0,00385 | 0,00311 | 0,00355 | 0,00251 | 0,00251 | 0,00251 | 0,00251 |
| 09_AN10 | 0,00366 |  | 0,00258 | 0,00370 | 0,00459 | 0,00325 | 0,00258 | 0,00254 | 0,00325 | 0,00258 | 0,00307 | 0,00258 | 0,00307 | 0,00258 | 0,00307 | 0,00258 | 0,00264 | 0,00325 | 0,00329 | 0,00260 | 0,00260 | 0,00307 | 0,00341 | 0,00258 | 0,00258 | 0,00258 | 0,00258 |
| 14_AN42 | 0,00366 | 0,00366 |  | 0,00278 | 0,00373 | 0,00186 | 0,00000 | 0,00247 | 0,00182 | 0,00000 | 0,00182 | 0,00000 | 0,00182 | 0,00000 | 0,00182 | 0,00000 | 0,00251 | 0,00186 | 0,00185 | 0,00263 | 0,00263 | 0,00182 | 0,00250 | 0,00000 | 0,00000 | 0,00000 | 0,00000 |
| 19_AN37 | 0,00737 | 0,00737 | 0,00366 |  | 0,00470 | 0,00322 | 0,00278 | 0,00369 | 0,00331 | 0,00278 | 0,00340 | 0,00278 | 0,00340 | 0,00278 | 0,00340 | 0,00278 | 0,00370 | 0,00322 | 0,00326 | 0,00372 | 0,00372 | 0,00340 | 0,00377 | 0,00278 | 0,00278 | 0,00278 | 0,00278 |
| 20_AN38 | 0,01110 | 0,01110 | 0,00734 | 0,01110 |  | 0,00430 | 0,00373 | 0,00462 | 0,00421 | 0,00373 | 0,00426 | 0,00373 | 0,00426 | 0,00373 | 0,00426 | 0,00373 | 0,00457 | 0,00430 | 0,00430 | 0,00459 | 0,00459 | 0,00426 | 0,00472 | 0,00373 | 0,00373 | 0,00373 | 0,00373 |
| 21_AN41 | 0,00550 | 0,00550 | 0,00182 | 0,00550 | 0,00921 |  | 0,00186 | 0,00320 | 0,00264 | 0,00186 | 0,00261 | 0,00186 | 0,00261 | 0,00186 | 0,00261 | 0,00186 | 0,00320 | 0,00000 | 0,00257 | 0,00325 | 0,00325 | 0,00261 | 0,00313 | 0,00186 | 0,00186 | 0,00186 | 0,00186 |
| 30_AN52 | 0,00366 | 0,00366 | 0,00000 | 0,00366 | 0,00734 | 0,00182 |  | 0,00247 | 0,00182 | 0,00000 | 0,00182 | 0,00000 | 0,00182 | 0,00000 | 0,00182 | 0,00000 | 0,00251 | 0,00186 | 0,00185 | 0,00263 | 0,00263 | 0,00182 | 0,00250 | 0,00000 | 0,00000 | 0,00000 | 0,00000 |
| 31_AN60 | 0,00366 | 0,00366 | 0,00366 | 0,00737 | 0,01110 | 0,00550 | 0,00366 |  | 0,00307 | 0,00247 | 0,00301 | 0,00247 | 0,00301 | 0,00247 | 0,00301 | 0,00247 | 0,00266 | 0,00320 | 0,00314 | 0,00351 | 0,00351 | 0,00301 | 0,00246 | 0,00247 | 0,00247 | 0,00247 | 0,00247 |
| 32_AN59 | 0,00550 | 0,00550 | 0,00182 | 0,00550 | 0,00921 | 0,00366 | 0,00182 | 0,00550 |  | 0,00182 | 0,00261 | 0,00182 | 0,00261 | 0,00182 | 0,00261 | 0,00182 | 0,00312 | 0,00264 | 0,00260 | 0,00335 | 0,00335 | 0,00261 | 0,00305 | 0,00182 | 0,00182 | 0,00182 | 0,00182 |
| 44_AN50P | 0,00366 | 0,00366 | 0,00000 | 0,00366 | 0,00734 | 0,00182 | 0,00000 | 0,00366 | 0,00182 |  | 0,00182 | 0,00000 | 0,00182 | 0,00000 | 0,00182 | 0,00000 | 0,00251 | 0,00186 | 0,00185 | 0,00263 | 0,00263 | 0,00182 | 0,00250 | 0,00000 | 0,00000 | 0,00000 | 0,00000 |
| 46_AN111 | 0,00550 | 0,00550 | 0,00182 | 0,00550 | 0,00921 | 0,00366 | 0,00182 | 0,00550 | 0,00366 | 0,00182 |  | 0,00182 | 0,00000 | 0,00182 | 0,00000 | 0,00182 | 0,00311 | 0,00261 | 0,00257 | 0,00315 | 0,00315 | 0,00000 | 0,00308 | 0,00182 | 0,00182 | 0,00182 | 0,00182 |
| 47_AN133 | 0,00366 | 0,00366 | 0,00000 | 0,00366 | 0,00734 | 0,00182 | 0,00000 | 0,00366 | 0,00182 | 0,00000 | 0,00182 |  | 0,00182 | 0,00000 | 0,00182 | 0,00000 | 0,00251 | 0,00186 | 0,00185 | 0,00263 | 0,00263 | 0,00182 | 0,00250 | 0,00000 | 0,00000 | 0,00000 | 0,00000 |
| 48_AN129 | 0,00550 | 0,00550 | 0,00182 | 0,00550 | 0,00921 | 0,00366 | 0,00182 | 0,00550 | 0,00366 | 0,00182 | 0,00000 | 0,00182 |  | 0,00182 | 0,00000 | 0,00182 | 0,00311 | 0,00261 | 0,00257 | 0,00315 | 0,00315 | 0,00000 | 0,00308 | 0,00182 | 0,00182 | 0,00182 | 0,00182 |
| 49_A133X | 0,00366 | 0,00366 | 0,00000 | 0,00366 | 0,00734 | 0,00182 | 0,00000 | 0,00366 | 0,00182 | 0,00000 | 0,00182 | 0,00000 | 0,00182 |  | 0,00182 | 0,00000 | 0,00251 | 0,00186 | 0,00185 | 0,00263 | 0,00263 | 0,00182 | 0,00250 | 0,00000 | 0,00000 | 0,00000 | 0,00000 |
| 50_AN136 | 0,00550 | 0,00550 | 0,00182 | 0,00550 | 0,00921 | 0,00366 | 0,00182 | 0,00550 | 0,00366 | 0,00182 | 0,00000 | 0,00182 | 0,00000 | 0,00182 |  | 0,00182 | 0,00311 | 0,00261 | 0,00257 | 0,00315 | 0,00315 | 0,00000 | 0,00308 | 0,00182 | 0,00182 | 0,00182 | 0,00182 |
| 53_AN20 | 0,00366 | 0,00366 | 0,00000 | 0,00366 | 0,00734 | 0,00182 | 0,00000 | 0,00366 | 0,00182 | 0,00000 | 0,00182 | 0,00000 | 0,00182 | 0,00000 | 0,00182 |  | 0,00251 | 0,00186 | 0,00185 | 0,00263 | 0,00263 | 0,00182 | 0,00250 | 0,00000 | 0,00000 | 0,00000 | 0,00000 |
| 56_AN54 | 0,00000 | 0,00366 | 0,00366 | 0,00737 | 0,01110 | 0,00550 | 0,00366 | 0,00366 | 0,00550 | 0,00366 | 0,00550 | 0,00366 | 0,00550 | 0,00366 | 0,00550 | 0,00366 |  | 0,00320 | 0,00320 | 0,00385 | 0,00385 | 0,00311 | 0,00355 | 0,00251 | 0,00251 | 0,00251 | 0,00251 |
| 60_AN93 | 0,00550 | 0,00550 | 0,00182 | 0,00550 | 0,00921 | 0,00000 | 0,00182 | 0,00550 | 0,00366 | 0,00182 | 0,00366 | 0,00182 | 0,00366 | 0,00182 | 0,00366 | 0,00182 | 0,00550 |  | 0,00257 | 0,00325 | 0,00325 | 0,00261 | 0,00313 | 0,00186 | 0,00186 | 0,00186 | 0,00186 |
| 62_AN77 | 0,00549 | 0,00549 | 0,00182 | 0,00549 | 0,00919 | 0,00365 | 0,00182 | 0,00549 | 0,00365 | 0,00182 | 0,00365 | 0,00182 | 0,00365 | 0,00182 | 0,00365 | 0,00182 | 0,00549 | 0,00365 |  | 0,00323 | 0,00323 | 0,00257 | 0,00306 | 0,00185 | 0,00185 | 0,00185 | 0,00185 |
| 64_AN87 | 0,00737 | 0,00366 | 0,00366 | 0,00737 | 0,01110 | 0,00550 | 0,00366 | 0,00737 | 0,00550 | 0,00366 | 0,00550 | 0,00366 | 0,00550 | 0,00366 | 0,00550 | 0,00366 | 0,00737 | 0,00550 | 0,00549 |  | 0,00000 | 0,00315 | 0,00346 | 0,00263 | 0,00263 | 0,00263 | 0,00263 |
| 66_AN96 | 0,00737 | 0,00366 | 0,00366 | 0,00737 | 0,01110 | 0,00550 | 0,00366 | 0,00737 | 0,00550 | 0,00366 | 0,00550 | 0,00366 | 0,00550 | 0,00366 | 0,00550 | 0,00366 | 0,00737 | 0,00550 | 0,00549 | 0,00000 |  | 0,00315 | 0,00346 | 0,00263 | 0,00263 | 0,00263 | 0,00263 |
| 69_AN68 | 0,00550 | 0,00550 | 0,00182 | 0,00550 | 0,00921 | 0,00366 | 0,00182 | 0,00550 | 0,00366 | 0,00182 | 0,00000 | 0,00182 | 0,00000 | 0,00182 | 0,00000 | 0,00182 | 0,00550 | 0,00366 | 0,00365 | 0,00550 | 0,00550 |  | 0,00308 | 0,00182 | 0,00182 | 0,00182 | 0,00182 |
| 70_AN58 | 0,00737 | 0,00737 | 0,00366 | 0,00737 | 0,01110 | 0,00550 | 0,00366 | 0,00366 | 0,00550 | 0,00366 | 0,00550 | 0,00366 | 0,00550 | 0,00366 | 0,00550 | 0,00366 | 0,00737 | 0,00550 | 0,00549 | 0,00737 | 0,00737 | 0,00550 |  | 0,00250 | 0,00250 | 0,00250 | 0,00250 |
| 73_AN41Q | 0,00366 | 0,00366 | 0,00000 | 0,00366 | 0,00734 | 0,00182 | 0,00000 | 0,00366 | 0,00182 | 0,00000 | 0,00182 | 0,00000 | 0,00182 | 0,00000 | 0,00182 | 0,00000 | 0,00366 | 0,00182 | 0,00182 | 0,00366 | 0,00366 | 0,00182 | 0,00366 |  | 0,00000 | 0,00000 | 0,00000 |
| 74_A112R | 0,00366 | 0,00366 | 0,00000 | 0,00366 | 0,00734 | 0,00182 | 0,00000 | 0,00366 | 0,00182 | 0,00000 | 0,00182 | 0,00000 | 0,00182 | 0,00000 | 0,00182 | 0,00000 | 0,00366 | 0,00182 | 0,00182 | 0,00366 | 0,00366 | 0,00182 | 0,00366 | 0,00000 |  | 0,00000 | 0,00000 |
| 79_AN72 | 0,00366 | 0,00366 | 0,00000 | 0,00366 | 0,00734 | 0,00182 | 0,00000 | 0,00366 | 0,00182 | 0,00000 | 0,00182 | 0,00000 | 0,00182 | 0,00000 | 0,00182 | 0,00000 | 0,00366 | 0,00182 | 0,00182 | 0,00366 | 0,00366 | 0,00182 | 0,00366 | 0,00000 | 0,00000 |  | 0,00000 |
| 83_AN89 | 0,00366 | 0,00366 | 0,00000 | 0,00366 | 0,00734 | 0,00182 | 0,00000 | 0,00366 | 0,00182 | 0,00000 | 0,00182 | 0,00000 | 0,00182 | 0,00000 | 0,00182 | 0,00000 | 0,00366 | 0,00182 | 0,00182 | 0,00366 | 0,00366 | 0,00182 | 0,00366 | 0,00000 | 0,00000 | 0,00000 |  |

The number of base substitutions per site from between sequences are shown. Standard error estimate(s) are shown above the diagonal. Analyses were conducted using the Kimura 2-parameter model [1]. The rate variation among sites was modeled with a gamma di

**References**

Kimura M. A simple method for estimating evolutionary rate of base substitutions through comparative studies of nucleotide sequences. J Mol Evol. 1980;16:111–20.

Tamura K, Stecher G, Kumar S. MEGA 11: Molecular Evolutionary Genetics Analysis Version 11. Mol Biol Evol. 2021. doi: https://doi.org/10.1093/molbev/msab120.

Stecher G, Tamura K, Kumar S. Molecular Evolutionary Genetics Analysis (MEGA) for macOS. Mol Biol Evol. 2020;37:1237–9.

**Supplementary Table S2. Haplotypes and accession numbers for sequences of the study**

| **Sample No** | ***Anopheles* species** | **Haplotype** | **NCBI accession number** | **included in Fig. 3** |
| --- | --- | --- | --- | --- |
| 64_AN87 | *An. funestus* | H1 | PQ789189 | x |
| 66_AN96 | *An. funestus* | H1 | PQ789189 | x |
| 09_AN10 | *An. funestus* | H2 | PQ789190 | x |
| 07_AN8 | *An. funestus* | H3 | PQ789191 | x |
| 56_AN54 | *An. funestus* | H3 | PQ789191 |  |
| 31_AN60 | *An. funestus* | H4 | PQ789192 |  |
| 70_AN58 | *An. funestus* | H5 | PQ789193 | x |
| 19_AN37 | *An. funestus* | H6 | PQ789194 |  |
| 46_AN111 | *An. funestus* | H7 | PQ789195 |  |
| 48_AN129 | *An. funestus* | H7 | PQ789195 |  |
| 50_AN136 | *An. funestus* | H7 | PQ789195 |  |
| 69_AN68 | *An. funestus* | H7 | PQ789195 |  |
| 21_AN41 | *An. funestus* | H8 | PQ789196 |  |
| 60_AN93 | *An. funestus* | H8 | PQ789196 | x |
| 14_AN42 | *An. funestus* | H9 | PQ789197 | x |
| 30_AN52 | *An. funestus* | H9 | PQ789197 | x |
| 44_AN50P | *An. funestus* | H9 | PQ789197 |  |
| 47_AN133 | *An. funestus* | H9 | PQ789197 |  |
| 49_A133X | *An. funestus* | H9 | PQ789197 |  |
| 53_AN20 | *An. funestus* | H9 | PQ789197 |  |
| 73_AN41Q | *An. funestus* | H9 | PQ789197 |  |
| 74_A112R | *An. funestus* | H9 | PQ789197 |  |
| 79_AN72 | *An. funestus* | H9 | PQ789197 |  |
| 83_AN89 | *An. funestus* | H9 | PQ789197 |  |
| 32_AN59 | *An. funestus* | H10 | PQ789198 | x |
| 20_AN38 | *An. funestus* | H11 | PQ789199 |  |
| 62_AN77 | *An. funestus* | H12 | PQ789200 |  |
| 76_AN47 | *An. funestus* | *NA** | PQ796096 | x |
| 01_AN9 | *An. gambiae* | H1 | PQ789201 |  |
| 03_AN6 | *An. gambiae* | H1 | PQ789201 |  |
| 05_AN6 | *An. gambiae* | H1 | PQ789201 |  |
| 08_AN9 | *An. gambiae* | H1 | PQ789201 |  |
| 15_AN40 | *An. gambiae* | H2 | PQ789202 | x |
| 59_AN95 | *An. gambiae* | H2 | PQ789202 |  |
| 84_AN80 | *An. gambiae* | H2 | PQ789202 | x |
| 86_AN71 | *An. gambiae* | H2 | PQ789202 | x |
| 13_AN33 | *An. gambiae* | H3 | PQ789203 | x |
| 35_AN20 | *An. gambiae* | H3 | PQ789203 |  |
| 52_AN139 | *An. gambiae* | H3 | PQ789203 |  |
| 28_AN53 | *An. gambiae* | H4 | PQ789204 |  |
| 37_AN53 | *An. gambiae* | H4 | PQ789204 |  |
| 16_AN14 | *An. gambiae* | H5 | PQ789205 | x |
| 18_AN34 | *An. gambiae* | H6 | PQ789206 | x |
| 38_AN47 | *An. gambiae* | H7 | PQ789207 | x |
| 81_AN88 | *An. gambiae* | H8 | PQ789208 |  |
| AN1_E08 | *An. rufipes* | H1 | PQ789209 | x |
| 22_AN32 | *An. rufipes* | H2 | PQ789210 |  |
| 45_AN12 | *An. rufipes* | H3 | PQ789211 | x |
| 51_AN100 | *An. rufipes* | H4 | PQ789212 | x |
| 55_AE19X | *An. rufipes* | H5 | PQ789213 | x |
| 78_AN80 | *An. rufipes* | H6 | PQ789214 |  |
| 27_AN26 | *Anopheles* sp. | *NA* | PQ789215 | x |

*Sequence contained an ambiguous base and was therefore not included in the haplotype network analysis

**Supplementary Table S3. Accession numbers and countries for reference sequences used in haplotype network analyses**

| ***Anopheles* species** | **Country** | **Accession number** |
| --- | --- | --- |
| *An. rufipes* | Guinea | KM097029 |
| *An. rufipes* | Japan | LC473604 |
| *An. rufipes* | Malawi | LC473605 |
| *An. rufipes* | Mali | MK586026 |
| *An. rufipes* | Mali | MK586031 |
| *An. rufipes* | Mali | MK586042 |
| *An. rufipes* | Mali | MK586047 |
| *An. rufipes* | Mali | MK586048 |
| *An. rufipes* | Mali | MK586049 |
| *An. rufipes* | Mali | MK586050 |
| *An. rufipes* | Mali | MK586036 |
| *An. rufipes* | Mali | MK586044 |
| *An. rufipes* | Mali | MK586046 |
| *An. rufipes* | Zambia | MK776735 |
| *An. rufipes* | Zambia | MK776736 |
| *An. rufipes* | Zambia | OK017054 |
| *An. rufipes* | Zambia | PP105076 |
| *An. rufipes* | Botswana | MT741515 |
| *An. rufipes* | Botswana | PP747847 |
| *An. rufipes* | Botswana | PP815715 |
| *An. rufipes* | South Africa | MW532834 |
| *An. rufipes* | Kenya | OM363234 |
| *An. rufipes* | Kenya | OM363250 |
| *An. rufipes* | Kenya | OM363256 |
| *An. funestus* | Kenya | KJ522832 |
| *An. funestus* | Kenya | KU187102 |
| *An. funestus* | Kenya | KU187103 |
| *An. funestus* | Kenya | KU187104 |
| *An. funestus* | Kenya | KU380404 |
| *An. funestus* | Kenya | MH299885 |
| *An. funestus* | Kenya | MH299886 |
| *An. funestus* | Kenya | MH299887 |
| *An. funestus* | Kenya | MH299888 |
| *An. funestus* | Kenya | MH299889 |
| *An. funestus* | Kenya | MH299890 |
| *An. funestus* | Kenya | MK300231 |
| *An. funestus* | Kenya | MK300232 |
| *An. funestus* | Kenya | MT375215 |
| *An. funestus* | Kenya | MT375216 |
| *An. funestus* | Kenya | MT375217 |
| *An. funestus* | Kenya | MT375218 |
| *An. funestus* | Kenya | MT375219 |
| *An. funestus* | Kenya | MT375220 |
| *An. funestus* | Kenya | OM363229 |
| *An. funestus* | Kenya | OM363248 |
| *An. funestus* | DRC | MH384970 |
| *An. funestus* | Zambia | MK016596 |
| *An. funestus* | Zambia | MK016627 |
| *An. funestus* | Gabon | MW603513 |
| *An. funestus* | CAR | OM630640 |
| *An. funestus* | CAR | OM630641 |
| *An. funestus* | CAR | OM630642 |
| *An. funestus* | Madagascar | OM630643 |
| *An. funestus* | Angola | OR839824 |
| *An. funestus* | Angola | OR839825 |
| *An. funestus* | Angola | OR839826 |
| *An. funestus* | Angola | OR839827 |
| *An. funestus* | Angola | OR839828 |
| *An. funestus* | Angola | OR839834 |
| *An. funestus* | Angola | OR839844 |
| *An. funestus* | Angola | OR839847 |
| *An. funestus* | Senegal | PQ104890 |
| *An. gambiae* | Liberia | KR152318 |
| *An. gambiae* | Togo | KR152328 |
| *An. gambiae* | Togo | KR152330 |
| *An. gambiae* | Ghana | LC507832 |
| *An. gambiae* | Kenya | KU187108 |
| *An. gambiae* | Kenya | KU187109 |
| *An. gambiae* | Kenya | MK300234 |
| *An. gambiae* | Kenya | MT375221 |
| *An. gambiae* | Kenya | MK300237 |
| *An. gambiae* | Kenya | MT375222 |
| *An. gambiae* | Kenya | MT375223 |
| *An. gambiae* | Kenya | MT375224 |
| *An. gambiae* | Uganda | MG753662 |
| *An. gambiae* | Uganda | MG753664 |
| *An. gambiae* | Uganda | MG753669 |
| *An. gambiae* | Uganda | MG753670 |
| *An. gambiae* | Uganda | MG753673 |
| *An. gambiae* | Uganda | MG753687 |
| *An. gambiae* | Uganda | MG753690 |
| *An. gambiae* | Uganda | MG753693 |
| *An. gambiae* | Uganda | MG753695 |
| *An. gambiae* | Uganda | MG753696 |
| *An. gambiae* | Uganda | MG753698 |
| *An. gambiae* | Uganda | MG753702 |
| *An. gambiae* | Uganda | MG753710 |
| *An. gambiae* | Uganda | MG753713 |
| *An. gambiae* | Uganda | MG753715 |
| *An. gambiae* | Uganda | MG753719 |
| *An. gambiae* | Uganda | MG753723 |
| *An. gambiae* | Uganda | MG753725 |
| *An. gambiae* | Uganda | MG753733 |
| *An. gambiae* | Uganda | MG753732 |
| *An. gambiae* | Uganda | MG753737 |
| *An. gambiae* | Uganda | MG753740 |
| *An. gambiae* | Uganda | MG753741 |
| *An. gambiae* | Uganda | MG753744 |
| *An. gambiae* | Uganda | MG753745 |
| *An. gambiae* | Uganda | MG753749 |
| *An. gambiae* | Uganda | MG753750 |
| *An. gambiae* | Uganda | MG753760 |
| *An. gambiae* | Uganda | MG753761 |
| *An. gambiae* | Uganda | MG753763 |
| *An. gambiae* | CAR | OM630646 |
| *An. gambiae* | Madagascar | OM630647 |
| *An. gambiae* | Madagascar | OM630649 |
| *An. gambiae* | Comoros | OP620446 |
| *An. gambiae* | OK236351 | Nigeria |
